# Obsessive-compulsive Symptoms Prevalence among Medical Students in Four Major Universities, Saudi Arabia: A Cross-Sectional Study

**DOI:** 10.2174/0117450179360311241216053222

**Published:** 2024-12-31

**Authors:** Feras A. Al-Awad, Kawther Hadhiah, Ahmad Albik, Ahmed Abdulwahab, Ali Almatiq, Ali Alhumran, Husain Alsaffar, Mohammed Alomar, Mohammed A. BuSaad

**Affiliations:** 1 Psychiatry Department, College of Medicine, Imam Abdulrahman Bin Faisal University, Dammam, Saudi Arabia; 2 Department of Neurology, College of Medicine, Imam Abdulrahman Bin Faisal University, Dammam, Saudi Arabia; 3 College of Medicine, Imam Abdulrahman Bin Faisal University, Dammam, Saudi Arabia; 4 Department of Family and Community Medicine, College of Medicine, Imam Abdulrahman Bin Faisal University, Dammam, Saudi Arabia

**Keywords:** Obsessive-compulsive disorder, Prevalence, Medical students, OCI-R scale, Health Awareness Programs, Obsessive-compulsive Symptoms

## Abstract

**Background:**

Obsessive-compulsive disorder is recognized by the World Health Organization as one of the top 10 most disabling disorders globally. Characterized by recurrent and persistent thoughts (obsessions) and/or repetitive behaviors (compulsions), it significantly disrupts an individual's daily life, impacting routine, education, career development, and social relationships. The disorder's prevalence varies worldwide, with studies in Saudi Arabia showing a higher rate of obsessive-compulsive symptoms among medical students.

**Objective:**

This study aimed to determine the prevalence of obsessive-compulsive symptoms among undergraduate medical students in four major universities in Saudi Arabia.

**Methods:**

A cross-sectional, web-based survey was administered between April and June 2023 to medical students from Imam Abdulrahman Bin Faisal University, King Faisal University, King Saud University, and King Abdulaziz University. The survey assessed obsessive-compulsive symptoms using the OCI-R scale.

**Results:**

Out of 886 medical students, 411 (46.4%) scored 21 or more on the OCI-R, indicating probable obsessive-compulsive disorder. Females had significantly higher OCI-R scores compared to males. Younger students, those with a family or personal history of psychiatric disorders, particularly depression, and those with lower academic satisfaction exhibited higher scores.

**Conclusion:**

This study emphasizes the importance of incorporating mental health awareness programs into educational curricula due to the high prevalence of obsessive-compulsive symptoms among medical students. Implementing such programs can help reduce stress and improve both academic performance and overall well-being.

## 1 INTRODUCTION

The World Health Organization (WHO) regards Obsessive-compulsive Disorder (OCD) as one of the top 10 most disabling disorders [1]. OCD is characterized by both obsessions and/or compulsions. Compulsions and obsessions can consume significant time, severely disrupting various aspects of life, including daily tasks, education, employment, career development, and social relationships [2].

According to The Diagnostic and Statistical Manual of Mental Disorders, Fifth Edition (DSM-5), obsessions are recurrent and persistent thoughts, urges, or images that are experienced as intrusive and unwanted. When experienced, such anxious thoughts can be relieved by performing some acts known as compulsions. Compulsions are repetitive behaviors or mental acts that an individual feels driven to perform according to rules that must be applied rigidly [3]. Obsessions are ego-dystonic, meaning that individuals recognize them as irrational thoughts that are inconsistent with their beliefs. They can involve many themes, including fear of contamination, symmetry, or religion. Compulsions, on the other hand, are unenjoyable acts that can be behavioral acts, such as checking whether the door is closed, or a mental act, like counting to a certain number [2]. Merely, the presence of obsessions and/or compulsions is not enough to establish a diagnosis of OCD. They must be time-consuming, causing marked distress, and interfering with one’s routine, functioning, or social life [3]. For example, in a study involving high school students, out of 2,323 participants, approximately 18.3% exhibited Obsessive-compulsive Symptoms (OCS). However, only 3.3% received a diagnosis of OCD [4].

The lifetime prevalence of OCD is estimated to be 2.3%. The mean age of onset of OCD is 19.5 years. Co-morbidities with other psychiatric disorders are highly common, affecting about 90% of individuals. The most common co-morbid conditions are anxiety disorders (75.8%), followed by mood disorders (63.3%) [5]. In Saudi Arabia, the lifetime prevalence of OCD is estimated to be 4.2%, with the mean age of onset being 16.8 years [6].

Medical students have a higher burden of psychological stress, depression, and anxiety compared to the general population in different countries. Many factors, including academic pressure, workload, sleep deprivation, and exposure to patients, have been hypothesized to contribute to poor mental health among medical students [7]. Studies conducted among medical students in Saudi Arabia have shown a higher prevalence of OCD compared to the general population. For instance, a study conducted in 2021 at Taibah University in Madina found that 26% of medical students had probable OCD [8]. Similarly, a research study conducted among medical students at Al Imam Mohammad Ibn Saud Islamic University in Riyadh in 2020 reported a prevalence of probable OCD at 36.2% [9]. Additionally, a study conducted in 2019 at Umm Al-Qura University on female students at the College of Applied Medical Sciences revealed a prevalence of OCS of 20%, with confirmed cases of OCD having a prevalence of 5.06% [10]. Given the heightened prevalence observed within the medical field, particularly among medical students, there is a pressing need for more extensive investigation. Therefore, this study aimed to explore the prevalence of OCS among undergraduate medical students in four major universities in the Kingdom.

## 2 METHODS

### 2.1 Study Design and Participants

A cross-sectional, web-based, self-administered survey was conducted across four major universities in Saudi Arabia: Imam Abdulrahman Bin Faisal University (IAU), King Faisal University (KFU), King Saud University (KSU), and King Abdulaziz University (KAU). The participants comprised medical students from all five years of medical school. Notably, in IAU and KAU, participants were from the second to the sixth year of medical school, excluding the first preparatory year. The survey was distributed between April and June 2023. It was conducted in English, reflecting the English language medium of instruction in medical schools in Saudi Arabia.

The study adhered to Good Clinical Practice (GCP) and met all relevant regulatory standards. Ethical approval was granted by the institutional review board at Imam Abdulrahman bin Faisal University (IRB-UGS- 2023-01-137) before initiating the survey. Participation was voluntary, and individuals received written informed consent through the web-based survey process.

### 2.2 Survey Development

A structured questionnaire was formulated following a comprehensive review of existing literature, incorporating necessary adjustments for the purpose of this study. The survey comprised four main sections, covering socio- demographic characteristics, medical background, academic performance, and assessments related to obsessive-compulsive symptoms (OCI-R).

The questionnaire collected demographic information, including gender (male, female), age in years (16, 17, 18, 19, 20, 21, 22, 23, 24, 25, 26, 27, 28, 29, 30, 31, 32), nationality (Saudi, non-Saudi), university (IAU, KFU, KSU, KAU), academic level (1^st^ year medical student, 2^nd^ year medical student, 3^rd^ year medical student, 4^th^ year medical student, 5^th^ year medical student), marital status (single, married, divorced, widowed), living situation (alone, with family, with friends/peers), and household income (<5000 SR, 5000-9999 SR, 10000-19999 SR, ≥20000 SR). It also addressed medical background by inquiring about prior diagnoses of psychiatric illnesses (no, yes) and family history of OCD (no, yes). Additionally, it explored academic factors, such as satisfaction with academic performance (very unsatisfied, unsatisfied, neutral, satisfied, very satisfied), adjustment to new academic years (I adjust easily to a new academic year, I have difficulties in adjusting to a new academic year, Not easy but not difficult), and repetition of any year (no, yes), thereby providing a comprehensive overview of participants’ backgrounds for the research.

To evaluate OCS, the study employed OCI-R, a shortened version of OCI, recognized as a valuable diagnostic screening tool in both research and clinical contexts [11]. With 18 items distributed across 6 subscales (washing, checking, neutralizing, obsessing, ordering, and hoarding), the OCI-R yields a maximum total score of 72, and a cutoff of 21 or more suggests probable OCD [12]. The OCI-R was employed based on evidence supporting its reliability and validity in assessing OCS in the Saudi Arabian population. A study conducted in Aseer Province confirmed its psychometric properties, establishing it as a reliable and valid tool for this context [13].

### 2.3 Survey Pretesting

Prior to initiating data collection, a pilot test of the survey was conducted among 20 medical students at IAU. Their feedback was utilized to evaluate the clarity, comprehensibility, and relevance of the survey questions to the study's objectives. The pilot study results informed adjustments to the survey and assessed the feasibility of the proposed plan for survey administration and data collection. Data from the pilot study were excluded from the main study.

### 2.4 Survey Administration

Before distributing the survey to potential participants, a minimum sample size of 366 was calculated using Epi Info, with a 95% confidence level and a 5% margin of error, based on the 2018/2019 count of medical students in Saudi Arabia from Statista [14]. Although proportional sampling suggested specific numbers from each university, the required number to be collected from each batch was less than 30. To make participant recruitment more convenient, we opted to use convenience sampling, aiming to collect at least 40 participants from each batch. This approach ensured sufficient representation while facilitating the data collection process, thus effectively covering the required sample size. It was deemed suitable given the web-based nature of the study and the need for timely data collection within a defined timeframe. However, the voluntary nature of the survey introduced the potential for self-selection bias, where students with specific characteristics (*e.g*., higher OCD) might be more likely to participate. Data collectors were recruited from all medical years in the four universities and were reached through social media platforms, such as WhatsApp, X (formerly known as Twitter), and Telegram. They were assigned unique identifying numbers and were instructed to distribute the survey to their respective batch *via* WhatsApp until a minimum of 40 participants were recruited, aiming to recruit an equal number of male and female medical students as much as possible.

Continuous monitoring and evaluation of the recruitment process guided subsequent adjustments to the data collection plan, particularly in identifying areas where targeted interventions were necessary for specific batches to meet their respective recruitment targets.

### 2.5 Data Analysis

All participants had complete sociodemographic, academic, and health-related data, and as such, they were all included in our analysis, constituting a total analytical cohort of 886 participants. For descriptive statistics, medians and Interquartile Ranges (IQRs) were estimated for continuous variables, while frequencies and proportions were used to characterize categorical variables.

The outcome of interest, OCI-R score, was presented as median and IQR, given its non-normal distribution. Two-sample Wilcoxon test or Kruskal-Wallis test were used to test significant differences in the medians of OCI-R score across the subgroups of the different sociodemographic, academic, and health-related variables. Multivariate linear regression was used to test the association between participants’ OCI-R score as the independent variable and the different variables controlled for in the model. The regression model was adjusted for potential confounders determined a priori using theoretical knowledge and prior literature. Factors adjusted for in the model included sociodemographic variables (age, gender, living situation, household income), academic variables (university, adjustment to new academic year, satisfaction with academic performance, repetition of any year), and health variables (family history of OCD and history of psychological illness). We also conducted an assessment of potential interactions between key variables, including gender and living situation, academic performance and satisfaction, and personal and family history of psychiatric illness, to explore their potential impact on the outcome of the OCI-R score. Analyses were performed using Stata^©^ software version 18 (StataCorp, College Station, TX).

## 3 RESULTS

Within the complete sample (n=886), gender distribution was nearly equal, with 48.9% male and 51.1% female representation (Table **1**). The predominant demographic characteristics included a high proportion of Saudi nationals (98.4%), unmarried individuals (96.4%), and those residing with their families (86.8%). Conversely, 9.3% lived independently, and a mere 4% shared residences with friends or peers. Regarding monthly household income, which was converted from Saudi Riyal to United States Dollar (USD), more than half of the participants reported incomes on the higher spectrum, with 26.1% indicating 2661-5323 USD and 39.2% reporting an income of ≥5323 USD. In terms of university affiliation, a notable percentage of medical students originated from KFU (29.5%) and IAU (27.7%), followed by KAU students (24.5%), and lastly, KSU (18.4%). Lastly, the prevalence of OCD among medical students, as determined by an OCI-R score of ≥21, was 46.4% (411 students), while 53.6% (475 students) scored below this threshold.

Table **2** shows the median OCI-R score and IQR across the subgroups of sociodemographic, academic, and clinical variables. The median OCI-R score for the overall sample was 19, with an Interquartile Range (IQR) of 11 to 32. Notably, the sociodemographic variables of nationality and marital status showed non-significant differences with p-values of 0.35 and 0.53, respectively, whereas living situation approached significance with a p-value of 0.06. On the other hand, there was a significant gender difference in OCI-R median score (*p* < 0.01) as females exhibited a higher median score (21, IQR: 12-34) compared to males (17, IQR: 10-30). As per household income, there was a significant association with a higher median OCI-R score shown by those belonging to the 1330-2661 USD income level (p = 0.01). Variables related to the university of study and academic life were all found to be significantly associated with the median OCI-R score (*p* < 0.05). The highest OCI-R scores were found among those studying at KAU and students who found it difficult to adjust to the new academic year with OCI-R scores of 22 (IQR: 13-35) and 24 (IQR: 14-36), respectively. Satisfaction with academic performance was inversely associated with the OCI-R score. Students who were very unsatisfied with their academic performance had the highest score (27.5, IQR: 19-36; *p*<0.01). Similarly, students with academic setbacks who needed to repeat any academic year had a higher OCI-R (24, IQR: 13-34.5; *p*=0.04). Lastly, participants with a family history of OCD and those who had a history of psychological illness reported significantly higher OCI-R scores (28, IQR: 15-38 and 24.5, IQR: 15-36), respectively.

In Table **3**, fully adjusted estimates of the multivariate analysis are displayed, examining the association between the different variables and the outcome of the OCI-R score. Age was inversely associated with the OCI-R score, decreasing to 1.92 for each additional year (95% CI: -2.42 to -1.41; *p* < 0.01). Females tended to have 1.86 more OCI-R units compared to male participants (95% CI: 0.13 to 3.59; *p* < 0.05). Living situation and household income seemed to have no significant influence on OCI-R scores, except for individuals earning 1330-2661 USD, as they showed an increase in OCI-R scores by 3.1 (95% CI: -1.06 to 7.62, *p* < 0.05) compared to individuals earning less than 1330 USD. Concerning university affiliation, it influenced OCI-R scores. Compared to students attending IAU, students from KSU and KAU showed significantly higher OCI-R scores by 2.8 (95% CI: 0.26 to 5.35; *p* < 0.05) and 5.54 (95% CI: 3.15 to 7.94; *p* < 0.01), respectively. Having “somewhat difficulty” in adjusting to the new academic year showed a significant decrease in the OCI-R score by -2.85 (*p*<0.01). As a gradient effect was observed, satisfaction with academic performance appeared to be a crucial factor. Increasing satisfaction was inversely associated with OCI-R score, where “very satisfied” students appeared to have the most significant decrease in OCI-R score by 8.59 units (95% CI: -12.71 to -4.47; *p* < 0.01). Lastly, a positive family history of OCD and prior psychological illness seemed to be positive contributors to increasing OCI-R scores by 3.76 (*p* <0.05) and 3.21 (*p* <0.01), respectively. Upon assessing the interaction between female gender and living situation, it was found that females living alone (compared to females living with family) had OCI-R scores that were, on average, 10.10 points higher (β = 10.10, 95% CI: 3.93–16.27, p = 0.001) compared to their counterparts.

## 4 DISCUSSION

This study aimed to assess the prevalence and factors associated with OCS among medical students across four major universities in Saudi Arabia. The findings revealed a considerable prevalence of OCS within this population, underscoring the heightened vulnerability of medical students to mental health challenges. This was also evident in a Canadian cross-sectional study showing that medical students had significantly higher rates of psychiatric disorders and psychological distress compared to the general population of the same age [15]. Out of the 886 medical students who participated in our study, 411 (46.4%) scored above 21, indicating probable OCD according to the OCI-R score. This prevalence has been found to be consistent with other studies performed at different medical colleges in Saudi Arabia [8, 9].

Our findings generally showed a higher prevalence compared to other studies conducted locally and internationally. A study at Taibah University in Saudi Arabia reported that 26% of participants had probable OCD using the OCI-R [8]. Another Saudi study in Riyadh found a prevalence of 36.2% [9]. In comparison, a study by Yoldascan *et al*. (2009) found a prevalence of OCD of 29.9% among Turkish university students [16]. Interestingly, a Brazilian study that applied a higher cutoff score (>27 points) reported a significantly lower prevalence of 3.8% [17]. Additionally, an earlier study from the United States, using a different scale (LOI-SF) with a cutoff of 28.4, found a prevalence of 5.2% among medical students [18]. The lower prevalence found by the two studies could be explained by the use of different cutpoints or scales; however, they both reported the prevalence to be significantly higher compared to the general population, indicating medical students to be at a high risk of developing OCD globally [17, 18]. The differences between populations could also be influenced by the various cultural factors, academic pressures, or educational environments across these populations.

Our study found a significant gender difference in the mean OCI-R score, as females averaged 1.86 points higher than males after adjusting for other factors (*p* < 0.05). Specifically, females had a mean score of 21, which reached the clinical threshold for OCD, compared to males, with a mean score of 17. This finding was consistent with a study conducted in Riyadh assessing the prevalence of OCD among medical students in which there was a statistically significant higher prevalence among females [9]. This may be probably due to the fact that female medical students are more prone to anxiety and depression, both closely tied to OCD [19]. It might be also related to hormonal fluctuations and the pressure to achieve perfectionism in medical school, further increasing their vulnerability to OCS. Contrastingly, other studies conducted in various countries have reported no significant gender difference, while some others have found a significant positive association between OCD and males [16, 17, 20, 21]. These findings suggest a large variation in OCD and OCS across different populations and demographics.

Although the mean age of onset of OCD is 16.8 years in Saudi Arabia, our study also revealed an inverse relationship between age and OCI-R score. This indicates that younger medical students may experience more pronounced OCS, possibly due to the stress of starting medical school. This decrease in the level of stress through the years was highlighted in a study assessing the prevalence of stress among medical students in Saudi Arabia [22]. This was supported by Taher *et al*., who also found OCS to be significantly related to younger age and earlier years of study among Iraqi medical students [23]. However, this contrasted with the results of Khalaf *et al*., Sultan *et al*., and Alsubaie *et al*., who did not find a significant age-related difference [9, 10, 13].

When looking at the relationship among OCD, age, and gender, the literature was found to show interesting patterns. Studies that looked at age of onset showed that OCS often occurs during childhood, with clinical OCD typically appearing in early adulthood [24]. The majority of cases were reported to occur by the age of 20, with few new cases after 30 years [25]. Age also appeared to relate to gender differences. Several childhood OCD studies reported a higher prevalence among males, while adult samples often exhibited equal or higher prevalence among females [25-27]. For example, a study in Switzerland reported a cumulative prevalence of 5.3% for females compared to 1.7% for males, and studies among adolescents found higher rates of OCS among females [27]. Such differences may stem from biological factors, such as hormonal influences, or sociocultural dynamics, including differing expressions of distress and societal expectations across genders. Moreover, males have often been noted to have a younger age of onset, associated with more severe and chronic presentations of OCD [28].

Furthermore, those with a family history of OCD showed a significantly higher mean OCI-R score. This was in line with the literature, suggesting OCD to have familiar aggregation and partially due to genetic factors. Studies on twins and families have been found to be compelling that genetics have a great impact on OCD [29]. For example, family studies have revealed a prevalence of 7%-15% of OCD in first-degree relatives. Also, twin studies have found concordance of 80% and 50% in monozygotic and dizygotic twins, respectively, suggesting OCD to be significantly impacted by genetics [30]. Additionally, this may also be attributed to behavioral modeling, where a child imitates his/her strict parents' behaviors and internalizes the high expectations placed upon him/her [31]. Such environments can reinforce obsessive-compulsive tendencies, as the child learns to adopt rigid patterns of behavior in an attempt to meet these demanding standards. However, two studies found no significant association between family history of OCD or psychiatric illness to OCD, possibly due to demographic or environmental differences, as well as a smaller sample size. In addition, a personal history of other psychiatric diseases was found to have a significant association with higher mean OCI-R scores. This finding was observed to align with numerous studies that have previously seen a significant relationship between OCD and depressive disorders and symptoms [10, 17, 32].

Another statistically significant variable associated with a higher mean OCI-R score was a household income of 1330-2661 USD compared to the reference group of <1330 USD. Interestingly, this was inconsistent with the existing literature, where several studies did not find a significant association between economic situation and OCD [17, 33]. In the Household Income and Expenditure Survey (2018) conducted by the General Authority for Statistics (GASTAT), the average monthly income of Saudi households was reported to be 3,950 USD [34]. This might suggest that the identified income range represents a segment of households earning below the national average, potentially reflecting unique socioeconomic factors specific to this group that could negatively influence OCD, warranting further investigation.

Participants in our study did not show a significant difference in OCS based on their living situation. This aligned with previous literature as Sultan (2021), Torres *et al*. (2016), and Özdemir (2016), all reported no significant association between OCS and living situation [10, 17, 20]. In contrast, Yoldascan *et al*. (2009) found significant associations between OCD and living in government dormitories, students’ or parental houses, and having a private room in the parental home [16]. In contrast to these broader patterns, our study highlighted a novel interaction: living situation can amplify the relationship between female gender and risk of OCD. Specifically, the impact of living alone, as opposed to living with family, was more pronounced among females than males, with the difference being 10 points higher for females when considering the interaction term (β = 10.10). This interaction highlighted that the dynamics of living alone may uniquely affect women. Also, this gender-specific interaction added to how living environments can shape mental health and suggested that targeted support strategies may be beneficial for female medical students.

Moreover, it was found that finding it somewhat difficult to adjust to the new academic year was significantly associated with lower OCI-R scores. Different results were found in the studies carried out by Torres *et al*. and Khalaf AM *et al*., although the association was not significant [17]. The options for this question in the survey were “easy”, “somewhat difficult”, and “difficult”, differing from the “yes” and “no” approaches used in these studies. This may explain the difference in results. It showed a significant association with lower OCI-R scores regarding satisfaction with academic performance. Similar, although not significant, results were found in the literature [9, 10, 17]. For this question, a scale of 5 options from “very unsatisfied” to “very satisfied” was used. It showed that OCI-R scores followed this scale, with higher scores for unsatisfaction and lower scores for satisfaction, and “very satisfied” showing the lowest score. This also differed from the “yes” and “no” approach used in the literature. Significant associations of higher OCI-R scores were also found with academic year repetition and being from KAU and KSU. However, these factors were not found to be explored in previous literature.

### 4.1 Strengths and Limitations

One of the strengths of our study was the inclusion of a large sample size drawn from multiple universities across Saudi Arabia, enhancing the representation of medical students compared to previous studies being limited to a single location or institution. Additionally, our focus on identifying the determinants of OCD provided practical insights that could enhance the usability of our findings and inform targeted prevention efforts in the future.

One limitation of this study was the potential variability in OCI-R scores due to differing academic schedules, as some students may have been in exam periods between April and June 2023, while others may not. Moreover, the voluntary nature of the survey introduced a risk of self-selection bias, as students with specific characteristics, such as higher levels of OCD, may have been more likely to participate. Although random sampling could have offered a more accurate representation, it was deemed impractical due to limitations in effectively reaching and obtaining responses from medical students across all four universities. In addition, the age of onset of OCS was not included in the questionnaire to support the relationship between OCS and medical school as these symptoms may have started prior to entering the medical school, which may have affected the applicability of the results to medical students.

As a next step, conducting interviews with students who scored above 21 on the OCI-R to assess whether they met the clinical criteria for OCD is recommended. This approach could offer a deeper understanding of the prevalence and characteristics of OCD among medical students. Future research should also consider employing random sampling to enhance the generalizability of findings and mitigate the selection bias present in this study. Given that academic pressure and seasonal variations likely influence OCS, longitudinal studies could provide valuable insights into the temporal dynamics of this relationship. Additionally, exploring specific academic stressors and comparing medical students with those in other disciplines could uncover unique challenges faced in medical education. Finally, assessing the impact of targeted mental health interventions within universities could inform the development of more effective support systems.

### 4.2 Implication

This study highlighted a high prevalence of OCS among medical students, with females, younger students, and those with a family history of OCD being more affected. Universities may enhance support by offering tailored mental health workshops and counseling for these groups. Integrating mental health education into the curriculum, conducting regular screenings, and promoting academic satisfaction through flexible grading, mentorship, and stress-reducing initiatives may improve both academic performance and mental well-being. Additionally, addressing challenges related to academic transitions with orientation programs and support networks could help reduce student stress.

## CONCLUSION

In conclusion, this study has highlighted a significant prevalence of OCS among medical students in Saudi Arabia, with nearly half of the participants showing probable OCD based on the OCI-R scale. Several factors have been identified as key predictors of OCS severity, including gender, age, family history of OCD, and psychiatric comorbidities. Female medical students have exhibited higher mean OCI-R scores than males, and younger students have reported more pronounced symptoms, indicating the need for targeted mental health support for these groups. Additionally, a family history of OCD and psychiatric illnesses has been significantly associated with higher OCS scores, reinforcing the genetic and environmental influences on OCD development. Academic satisfaction has also emerged as an important factor, with lower OCI-R scores observed among students being more satisfied with their academic performance. This finding has highlighted the potential link between academic well-being and mental health outcomes, emphasizing the need for interventions that support both academic and emotional health. While living situations have not shown a significant association with OCS in our sample, difficulties adjusting to a new academic year and repeating an academic year have been associated with higher OCI-R scores.

## Figures and Tables

**Table 1:** Baseline sociodemographic characteristics of study participants (n=886).

-	**Median (IQR) or n (%)**
**Characteristic**	**Total (n=886)**
**Age (years)**	22 (20, 23)
**Gender**	
Male	433 (48.9)
Female	453 (51.1)
**Nationality**	-
Saudi	872 (98.4)
Non-Saudi	14 (1.6)
**Marital status**	-
Single	854 (96.4)
Married	32 (3.6)
**Living Situation**	
With family	769 (86.8)
With friends/peers	35 (3.9)
Alone	82 (9.3)
**Household income (monthly)**	
<1330 USD	184 (20.8)
1330-2661 USD	124 (14.0)
2661-5323 USD	231 (26.1)
≥5323 USD	347 (39.2)
**University**	
IAU	245 (27.7)
KFU	261 (29.5)
KSU	163 (18.4)
KAU	217 (24.5)
**OCI-R (≥21)**	
Yes	411 (46.4)
No	475 (53.6)

**Note: **IAU: Imam Abdulrahman Bin Faisal University, KFU: King Faisal University, KSU: King Saud University, KAU: King Abdulaziz University. OCI-R: Obsessive Compulsive Inventory–Revised.

**Table 2:** Continous OCI-R score as median (IQR) across sociodemographic, academic, and medical subgroups (n=886).

**Characteristic**		-	**Median (IQR)**	***p*-value ^a^**
**OCI-R**		-	19 (11 – 32)	NA
**Gender**		Male	17.0 (10.0 - 30.0)	< 0.01
		Female	21.0 (12.0 - 34.0)	
**Living situation**		With family	19.0 (11.0 - 31.0)	0.06
		With friends/peers	24.0 (17.0 - 38.0)	
		Alone	19.0 (11.0 - 34.0)	
**Household income** **(monthly)**		<1330 USD	19.0 (12.0 - 32.5)	0.01
		1330-2661 USD	26.0 (12.0 - 36.0)	
		2661-5323 USD	19.0 (10.0 - 33.0)	
		≥5323 USD	17.0 (10.0 - 27.0)	
**University**		IAU	17.0 (10.0 - 30.0)	0.01
		KFU	19.0 (9.0 - 30.0)	
		KSU	19.0 (12.0 - 31.0)	
		KAU	22.0 (13.0 - 35.0)	
**Adjustment to new** **academic year**		Easy	18.0 (10.0 - 31.0)	< 0.01
		Somewhat difficult	17.0 (10.0 - 27.0)	
		Difficult	24.0 (14.0 - 36.0)	
**Satisfaction with** **academic performance**		Very unsatisfied	27.5 (19.0 - 36.0)	< 0.01
		Unsatisfied	22.0 (12.0 - 36.0)	
		Neutral	20.0 (10.0 - 32.0)	
		Satisfied	17.0 (9.5 - 28.0)	
		Very satisfied	15.0 (7.0 - 26.0)	
**Repeated any year**		No	19.0 (11.0 - 31.0)	0.04
		Yes	24.0 (13.0 - 34.5)	
**Family history of OCD**		No	19.0 (11.0 - 31.0)	< 0.01
		Yes	28.0 (15.0 - 38.0)	
**History of psychological illness**		No	18.0 (10.0 - 31.0)	< 0.01
		Yes	24.5 (15.0 - 36.0)	

**Note: **a: Wilcoxon two-sample test or Kruskal-Wallis test.

**Table 3:** Adjusted estimates of the multivariate analysis.

**Variable**	**Adjusted β**	**95% CI**	***p*-value**
**Age (years)**	-1.92	-2.42, -1.41	<0.01
**Gender**	-	-	-
Male	Reference	-	-
Female	1.86	0.13, 3.59	<0.05
**Living situation**	-	-	-
With family	Reference	-	-
With friends/peers	3.28	-1.06, 7.62	>0.05
Alone	0.47	-2.46, 3.39	>0.05
**Household income (monthly)**	-	-	-
<1330 USD	Reference	-	-
1330-2661 USD	3.10	0.20, 6.00	<0.05
2661-5323 USD	0.97	-1.52, 3.46	>0.05
≥5323 USD	-0.69	-3.04, 1.66	>0.05
**University**	-	-	-
IAU	Reference	-	-
KFU	1.78	-0.46, 4.02	>0.05
KSU	2.80	0.26, 5.35	<0.05
KAU	5.54	3.15, 7.94	<0.01
**Adjustment to the new academic year**	-	-	-
Easy	Reference	-	-
Somewhat difficult	-2.85	-4.93, -0.77	<0.01
Difficult	0.37	-2.04, 2.77	>0.05
**Satisfaction with academic performance**	-	-	-
Very unsatisfied	Reference	-	-
Unsatisfied	-2.28	-5.93, 1.38	>0.05
Neutral	-4.50	-8.09, -0.91	<0.05
Satisfied	-6.80	-10.49, -3.12	<0.01
Very satisfied	-8.59	-12.71, -4.47	<0.01
**Repeated any year**	-	-	-
No	Reference	-	-
Yes	2.76	-0.45, 5.97	<0.1
**Family history of OCD**	-	-	-
No	Reference	-	-
Yes	3.76	0.63, 6.90	<0.05
**History of psychological illness**	-	-	-
No	Reference	-	-
Yes	3.21	0.85, 5.57	<0.01

**Note: **The model was adjusted for age, gender, living situation, household income, university, adjustment to a new academic year, satisfaction with academic performance, repetition of any year, family history of OCD, and history of psychological illness.

## Data Availability

The authors confirm that the data supporting the findings of this research are available within the article.

## LIST OF ABBREVIATIONS

WHO: = World Health Organization
OCD: = Obsessive-compulsive Disorder
OCS: = Obsessive-compulsive Symptoms
DSM-5: = Diagnostic and Statistical Manual of Mental Disorders, Fifth Edition
OCI-R: = Obsessive-compulsive Symptoms
IQRs: = Interquartile Ranges

## STANDARDS OF REPORTING

STROBE guidelines were followed.
